# Autonomous Exploration for Radioactive Hotspots Localization Taking Account of Sensor Limitations

**DOI:** 10.3390/s19020292

**Published:** 2019-01-12

**Authors:** Hadi Ardiny, Stefan Witwicki, Francesco Mondada

**Affiliations:** Robotic Systems Laboratory, École Polytechnique Fédérale de Lausanne, CH-1015 Lausanne, Switzerland; witwicki@gmail.com (S.W.); francesco.mondada@epfl.ch (F.M.)

**Keywords:** autonomous exploration, sensor limitations, sensing systems, remote sensors, radioactive hotspots detection, safety

## Abstract

Effective radioactive hotspot localization and detection is limited by sensor characteristics (i.e., the long acquisition time and poor angular resolution AR of a gamma camera) that significantly degrade the performance of autonomous exploration in terms of the completion time and accuracy. The goal of this research is to study effective exploration algorithms that take into account these specific sensor limitations. These exploration algorithms are adapted and implemented based on behaviour-based and multi-criteria decision making MCDM approaches on an autonomous robot. The algorithms were also tested in simulation and validated by experiments performed on a real robot. According to the results, the algorithms demonstrate the ability to mitigate the unfavourable effects of the limitations.

## 1. Introduction

The meltdown of the Chernobyl nuclear power plant began on 26 April 1986 and was the worst civilian nuclear accident in history. Thirty-one of 237 people who had acute radiation sickness died within the first three months [1]. Several thousand fatal cancers were reported among the people who were in the contaminated areas in the years following the accident. The accident showed the world that it must ready to save human lives and decrease potentially serious radioactive effects. In the event of a nuclear accident, rescue robots can be used as an intervention tool to mitigate the damage. An important task for rescue robots is to map and find elements (e.g., victims, hazardous materials) inside a structure, in caves, or out in open areas. These robots are expected to quickly accomplish exploration without increasing risks such as additional collapses, damage, and hits to humans [2]. The post-disaster area is usually a threat to human life and long-term health, limiting rescuers from being in the zone for too long or even at all. Robots can safely explore such areas and provide vital information about the situation. For instance, robots can collect data about radioactive hotspots and victims’ situations and then plot this information on a global map. Based on this information, rescuers can decide what the best way is to save lives (e.g., a victim that has been exposed to high doses of radiation has to be removed or has to be isolated sooner than others).

Accordingly, the scenario we considered for our research was an environment after a nuclear disaster. The autonomous robots used for this situation were designed for exploration, having gamma cameras for radioactive hotspot detection and a laser range scanner to map the environment. The main contribution of this research is to adapt and study methods for an efficient exploration using autonomous robots that must work under gamma camera limitations. Even though much progress has been made in gamma camera technologies, there are still limitations that stifle their potential in exploration applications. The first limitation is the relatively long acquisition time of a gamma camera when preparing a radiation image; this is the main challenge for real-time exploration. The second limitation is poor angular resolution (AR). The poor AR makes it impossible to precisely and quickly localize radioactive hotspots that are placed far from a robot by use of a single image. Therefore, exploration algorithms are needed for the detection and localization of radioactive hotspots in a reasonable amount of time to accompany the mapping of the unknown environment. Behaviour-based and multi-criteria decision making (MCDM) approaches are two different methods used for autonomously performing exploration. We adapted these exploration methods to include the limitations of the gamma camera to study how efficient these approaches are for rescue purposes after a nuclear disaster.

## 2. Related Work

In this section, we survey the exploration strategies that have been presented in the literature. We cover both papers that were devoted to exploration strategies and papers in which systems were used to localize radioactive hotspots. In Section 2.1, we address exploration strategies that could be used in a rescue situation, and then, in Section 2.2, we investigate more specifically the methods that were used to detect and localize radioactive hotspots.

### 2.1. Exploration Strategies

In this section, we review the most significant and relevant research on exploration strategies that could be used in the rescue operation, from the simplest to the most complex. Exploration strategies can be roughly categorized into four groups: (1) strategies in which robots follow predefined paths; (2) robots move randomly; (3) strategies that direct robots based on reactive approaches; and (4) strategies that are based on deliberative and optimization methods.

In the first category, robots move on predefined trajectories. For instance, after a mine disaster, rescue teams attempt to receive information by sending the robot along a fixed path or a canal [3]. In [4], the robot follows several patterns as follows: SeedSpreader, Concentric, FigureEight, etc. These exploration paths were followed to construct visual maps. In [4], robots built an integrated map by moving on a predefined trajectory that was similar to an Archimedes spiral. In [5], a robot moved a fixed distance from its starting position. McGee and Hedrick [6] studied an exploration method for finding mobile intruders or evaders. A search robot was equipped with a special sensor to detect the evader within the sensor region. Without any assumption about the motion of the evader, the robot could start to cover the region by following a predefined trajectory. Aerial robots equipped with cameras perform assessments and surveillance of the environment by moving along predefined trajectories. For instance, an unmanned aerial vehicle (UAV) can assist rescuers in sweeping through regions looking for victims by analyzing human appearances or movements [7,8]. In [9], a UAV patroled a region along a predefined trajectory to detect fire in forests for firefighting. In [10], aerial robots with cameras on board were used to inspect surfaces.

In the second category, an exploration robot moves randomly. In [11] a robot moved randomly to explore an environment; this random walk is biased toward a frontier region. Arkin and Diaz [12] presented an anchored random exploration strategy that did not use any environmental information. The robots move randomly within their communication networks. Another application for random exploration is foraging: a large number of individuals search the environment, find targets (e.g., food), and then transport the targets to one or more depot sites [13]. For instance, multiple robots randomly explored an unknown environment in search of a specific food location and then carried the food to the hive location [14,15]. Search and rescue, landmine clearance, and harvesting are diverse real-world applications for foraging robots.

The third category shows exploration strategies that are based on reactive approaches. In reactive approaches, robots respond very quickly to changes in an environment [16]. Robots locally sense the environmental features and then act immediately according to defined rules. These rules can be a set of behaviours that are activated based on inputs and internal states. Behaviour-based exploration strategies are typically considered extensions of reactive approaches, and they are between reactive and planner-based approaches [17]. Schmidt et al. [18] defined different behaviours (i.e., obstacle avoidance and wall following) necessary to explore environments and then build topological maps. Van Nguyen et al. [19] introduced a new behaviour-based method for navigating the mobile robot in unknown environments. In this method, each behaviour was implemented by a fuzzy controller and executed independently. Cepeda et al. [20] used a behaviour-based navigation that was coupled with a memory that stored previously visited regions. This allowed robots to safely and efficiently explore the environment.

The purpose of autonomous exploration is typically to efficiently complete an operation within a reasonable amount of time, so some research has addressed a range of different deliberative perspectives. Some exploration systems evaluate exploration point candidates and then direct robots to the best point. Choosing the next target point can significantly improve performance of autonomous exploration. Yamauchi [21] introduced the frontier approach to explore an unknown environment. Frontier points are selected on the boundary that divides explored from unexplored space, and then robots choose destination points based on defined rules–for instance, selecting the nearest point to the current position of the robot. By following such an approach, exploration strategies could obtain more accurate information and minimize travel costs by optimizing exploration. These kinds of exploration strategies define utility functions to choose the next point. Burgard et al. [22] presented a utility function that simultaneously took into account the cost of reaching an unexplored location and the benefit acquired from doing so. Auat Cheein et al. [23] showed an algorithm to generate a harvesting schedule based on the priority queue together with the harvesting rate and the robot’s payload capacity. Wirth and Pellenz [24] presented an approach that took into account the distance to the next frontier and the difficulty of the robot’s path. The difficulty could be having to cross a narrow path or being in an open space where the sensors could not detect landmarks, among many other challenges. Then, the robot chose the safest path with the lowest cost to reach the target point. In another exploration approach [25], the decision was made based on the next best view (NBV). This means that the exploration system drove the robot to discover more information when it reached the target location.

Some exploration strategies based on utility functions take into account several criteria. In these strategies, the utility of a point is a combination of different criteria (e.g., travelling cost, total number of sensing operations, total number of stops to reach location, etc.), so the selected point with the highest utility value is the one that satisfies the most criteria. Tovar et al. [26] proposed an efficient exploration algorithm in which the utility function combined several criteria in a multiplicative form to evaluate candidate frontier points. Amigoni and Gallo [27] proposed a more general way to choose the best candidate location. In this multi-objective method, the values of the involved factors were kept separate from each other without combining them in a utility function; hence, a set of nondominated points (Pareto frontier) could be selected. Basilico and Amigoni [28] implemented a method based on MCDM for robots employed in search and rescue applications. In this research, a robot started to find targets (e.g., victims) in an unknown environment. One feature of this MCDM was that it considers dependency between criteria because of the interactions between them (e.g., travel cost and battery life). A multi-robot system was designed for real-time exploration [29]. The concept behind this work was to simultaneously explore and create a communication network through a chain of robots (for communication between robots). Some robots looked for victims and mapped the environment while other robots acted as relay terminals between the explorer robots and the base station. Each explorer robot selected the next target location among frontier points using a utility function.

### 2.2. Radioactive Hotspot Detection and Localization

In this section, we categorize the research of radioactive hotspot detection and localization according to the robots’ detectors as follows: non-directional (e.g., NaI scintillation detectors [30]) and directional (e.g., a gamma camera).

The non-directional detectors were often used to map environments in terms of radiation intensity, or a network of embedded non-directional detectors was used to find stationary or mobile sources. In [31,32,33], a network of fixed radiation sensors was used to identify radioactive stationary or mobile sources amidst background radiation. Baidoo-Williams [34] has defined the smallest number of fixed sensors to theoretically localize sources based on their gamma-ray count.

In contrast, for applications such as rescue operations, mobile detection systems are needed to search in post-disaster environments. In [35], an aerial robot was equipped with a non-directional radiation sensor; it was tasked with effectively mapping the whole environment, providing a radiation map, and then localizing strong radioactive sources.

In [36], UAVs cooperate to discover the gradient of the radiative intensity of a contaminated region. The locations of radioactive sources could be also extracted with additional processes onto a radioactive map [37].

Directional detectors (e.g., the CZT Compton camera) were used in much related research. Most of them obtain 2D directional images that used stationary detector systems [38,39,40]. In other research, two fixed-coded aperture cameras were used to estimate the spatial coordinates of a radioactive source by employing triangulation methods [41]. In this research, the sources were placed far from the detector in respect to the detector size.

In opposite to stationary detector systems, a detection system (an array composed of 18 CZT detectors) and a positioning system were placed on top of a cart to localize two radioactive sources [42]. The detection system was manually moved, and its position was recorded to reconstruct a radiation image in continuous motion. It could localize the point sources within 10 cm of the true source position after 4.5 min. Although authors presented a mobile exploration based on a series of data from a detection system (an array composed of 18 CZT detectors), it did not really perform autonomous exploration. Christian et al. [43] presented the RICA robot; it was a remote mobile platform equipped with a set of different radioactive detection system. The RICA robot was developed to carry out inspection and sampling missions in extremely dangerous sites. This robot could locate and measure the activity of radioactive sources by remote and in situ operations. The AISense [44], given in Table 1 with a 0.1 s acquisition time, seems to be a good choice for radioactive hotspot localization in autonomous exploration, but this detector could not detect radioactive sources separately if there was more than one of them in the environment. In the case of more than one source, the device assumes that it is one hotspot only and determines its location as being somewhere between the two real sources (i.e., a centre of mass of sources), depending on the individual activities of the hotspots and distances to each. As soon as the robot moves closer to one of them, this hotspot will take precedence over the other. This behaviour causes an inefficient exploration and can inhibit the discovery of all radioactive sources.

## 3. Problem Statement

In accordance with the literature, Section 2.2, non-directional detectors are inappropriate for rescue exploration because robots must scan the entire environment for mapping and finding radioactive hotspots, making it an extremely time-consuming choice. Directional detectors seem a better way but their limitations strongly confine autonomous exploration, especially where when efficiency is necessary, like performing rescue tasks. Moreover, autonomous exploration research which has been presented in Section 2.1 has addressed different autonomous exploration strategies without considering the detector limitations. To the best of our knowledge, there is not any specific research on autonomous exploration that has addressed the detector limitations. The aim of our research is to study efficient and autonomous exploration strategies for a mobile detection system composed of directional detectors with long acquisition time and poor AR limitations.

Typically, the problem with using a tardy detector such as a gamma camera is that a robot needs to stop many times to obtain the required information. The question is how and where must a robot stop to do so? To answer this question, we need an algorithm to drive the robot so that the robot stops only at optimal points for a few minutes (i.e., the acquisition time) to acquire as much information about the radioactive data as possible while concurrently making progress in its exploration. The second limitation of gamma cameras is that they usually have poor AR. This prevents precise localization of distant radioactive hotspots by a single image. Therefore, the robot needs to move toward the possible sources for better estimations of them.

In conclusion, we require exploration algorithms that can accomplish the exploration tasks as quickly as possible and that are confined to the characteristics of the gamma camera. In this research, we present two different exploration methods to address the problems of autonomous exploration when using a gamma camera for a rescue situation.

## 4. Exploration Approaches

Rescue robots need different sensors such as sonar, a laser range scanner, and cameras to map and provide a world model of required elements. In our scenario, a robot composed of a gamma camera, vision camera, and laser range scanner was designed to map and build a world model of elements (e.g., radioactive sources). We study behaviour-based and MCDM approaches that are considered for exploring an environment and recognizing elements after a nuclear disaster. As previously discussed, in addition to the time constraint for the rescue operation, we deal with two main limitations connected to the gamma camera’s characteristics. The proposed algorithms are adapted to address the sensor limitations’ challenges for performing an effective exploration.

### 4.1. The Behaviour-Based Approach

**Figure 1:**
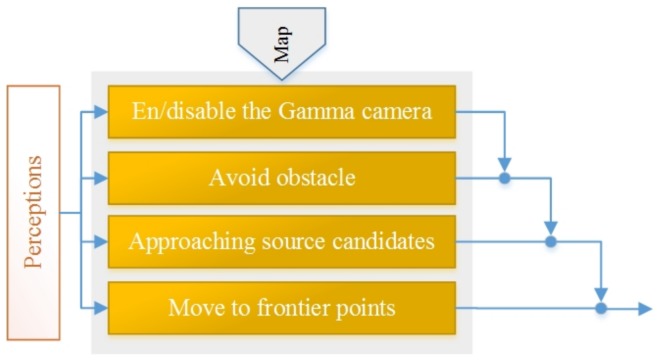
The control architecture for the behaviour-based system that performs exploration with limited detectors.

This section presents a behavioural approach employed for autonomous exploration with limited detectors. As presented in Section 2, the behaviour-based approach is one strategy used for autonomous exploration. In this paper, the behaviour-based approach is coupled with frontier path planning to address generic issues of autonomous exploration [49]. In addition, it is adapted for radioactive hotspot detection under the sensor limitations. Figure 1 shows the behaviour-based system consists of four behaviours that are described based on their priority:

*En/disable the gamma camera*

Because of the acquisition time limitation, the robot must stop for a certain amount of time to obtain a single radiation image. Determining where the robot should stop is the first issue of using this approach, and it impacts exploration performance. Our rule is to stop the robot wherever a defined amount of the gamma camera coverage area has not already been covered. The robot is constantly checking how much area the gamma camera has already covered, and if it is less than a defined threshold, the robot stops to record a new radiation image.

*Avoid obstacles*

This protective behaviour allows a robot to detect and avoid obstacles. Although using path planner methods based on the built map avoid the static obstacles, this behaviour is designed for unseen and dynamic obstacles. A robot detects close obstacles simultaneously and then changes direction or/and linear speed to avoid collisions. The behaviour design is based on the artificial potential field concept [50].

*Approaching source candidates*

The second limitation of the gamma camera is poor AR that detracts from precisely detecting and localizing the radioactive sources. In the defined rule for this behaviour, the robot must move nearer to radioactive source candidates to improve their locations. After the new radiation image is attained, false hotspots may be estimated somewhere around real radioactive sources because of poor AR. Therefore, the robot moves toward the nearest candidate radioactive hotspots to approve or discard them based on the method presented in Section 4.3. The robot continues its job within a defined loop until there are no more candidate points.

*Move to frontier points*

The frontier algorithm produces points on the boundary between free cells and unexplored areas. It aims to extend the map into new territories by choosing one of these points [21]. Therefore, this behaviour directs the robot to the next frontier point based on the optimal path to extend the map.

### 4.2. MCDM Approach

The MCDM approach as an exploration method is presented in Section 2.1 (related work), and it was designed to efficiently drive a robot by means of an effective decision in each step. In this section, we adapt the MCDM approach that takes into account the sensors’ limitations in performing an effective autonomous exploration. We define a global utility function that takes into account several criteria and some of these criteria are associated with sensors’ limitations. The core of this type of exploration method was to formulate a utility function based on the criteria defined by a designer. The utility function then evaluates all available candidates to present the next target (i.e., point or task) with the highest utility value. The selected target is the eligible candidate satisfying the most criteria. An action based on a sequence of selected points that have the highest utility values results in an effective exploration. In the next sections, we present the criteria, utility functions and describe how the MCDM approach is implemented.

#### 4.2.1. The Criteria

The idea behind the exploration method is to incrementally explore the environment by choosing the best point at each step according to the criteria. The criteria are defined in such a way as to include important factors that impact the exploration progress considerably. We define the criteria of our case for candidate points below.

*Detectable areas*

Most exploration strategies were developed to discover environments and build maps in the shortest amount of time. Therefore, if we already know how much area will be discovered when the robot reaches the next point, we can optimize the exploration by choosing the points with the highest gain. The detectable areas (Sd) show the amount of information gained from maps when the robot is on the next point.

In our scenario, exploration is required to collect different data and to build related maps; the robot operates on both obstacle maps and radiation covering maps. The obstacle map is an occupancy grid map that represents the environment with an array of cells; the value of each cell represents the probability of it being occupied by an obstacle. The radiation covering map is likewise composed of an array of cells, but binary values are assigned to these cells. The value of a cell becomes one if it has fallen within the coverage area of a detector. A binary map is a good choice for the covering map because the purpose of the radiation map is only to show how much of the environment has been mapped for radioactive hotspots. The gamma camera detects hotspots and then the position of radioactive sources is marked on a main layer of the semantic world model (See Section 5.).

The obstacle map is often probabilistic and a particular metric is required to measure the detectable area. We compute the entropy of each cell of a detectable area according to information theory (Shannon information). Equation (1), as proved by Cover and Thomas [51], shows how to compute the detectable area (Sd):

(1) $$H\left(cell_{i}\right)=\left\{\begin{array}{cc} 0, & \mathrm{if}\;Pr(cell_{i})=1\\ 0, & \mathrm{if}\;Pr(cell_{i})=0\\ (Pr\left(cell_{i}\right)\times log_{2}\left(\frac{1}{Pr(cell_{i})}\right)+\\ \left(1-Pr\left(cell_{i}\right)\right)\times log_{2}\left(\frac{1}{1-Pr(cell_{i})}\right), & \mathrm{otherwise} \end{array}\right.$$

(2) $$Sd=\sum_{cell_{i}\in C}H\left(cell_{i}\right)$$

where $cell_{i}$ is the number of cell *i*; $Pr(cell_{i})$ is a probability value that represent occupancy status of that cell and it comes from a probabilistic map; *H* is the entropy value for each cell; *C* is the coverage area of the sensor.

We have the two maps, and we need to estimate the potential extension of each map for candidate points. $Sd_{radiation}$ and $Sd_{obstacle}$ will be information gained about new discovered regions for radiation covering map and obstacle map, respectively, when the robot reaches the next point. The obstacle map is only a probabilistic map and each cell of this map has a value between zero (i.e., free cell) and one (a cell that is occupied by an obstacle); thus, Equation (1) is used to compute $Sd_{obstacle}$. The radiation covering map is composed of binary maps, and each cell is either zero or one. A cell will be equal to one if it is discovered by the detector. Therefore, we just count the cells with zero values within the coverage area of the related sensor to compute $Sd_{radiation}$ and to know how much the radiation covering map will be extended.

*Distance*

A common factor for optimizing exploration is the Euclidean distance (*d*) between the current position of the robot and a target point. It would be best to compute the shortest path between the robot and a target point based on the obstacle map while this path takes into account obstacles and walls. However, finding shortest paths based on the obstacle map for many points could drastically raise the computation time and effort, so, the Euclidean distance is considered.

*The total of possibilities*

Each radioactive source candidate has a variable that indicates the probability of it being a real element, and we called it possibility (See Section 4.3). This sounds reasonable enough if we consider possibility to be one criterion. This criterion can direct robots toward candidates that are more likely to be real elements. $U_{tos}$ indicates the sub-utility associated with this criterion.

*Number of possible real elements*

If a robot can approve the element (e.g., sources) quickly, false candidates made from the approved elements (because of poor AR or inaccuracies in the positioning system) will be removed. Obviously, having fewer candidates causes the exploration method to progress more quickly. $U_{pr}$ is the sub-utility for the number of real elements that can be approved.

#### 4.2.2. Definition of the Utility Function

The exploration method directs the robot to map and localize elements within a limited amount of time by selecting a point with the highest utility value. Utility values are computed with a global utility function that consists of different criteria such that each criterion is associated with one important factor in the exploration scenario.

To combine the criteria for measuring utility values, we normalize the sub-utilities linked to the criteria to the common scale I = [0, 1]. The target is selected only in the present situation of the environment for all available points at any step. Hence, we can normalize a sub-utility of a point with respect to the maximum and minimum values of the present set of points (*P*) as follows [52]:

(3) $$\hat{U}\left(p\right)=\frac{U\left(p\right)-\min_{\forall q\in P}\left(U\left(q\right)\right)}{\max_{\forall q\in P}\left(U\left(q\right)\right)-\min_{\forall q\in P}\left(U\left(q\right)\right)}\;\;\;\;\;p\in P$$

where *U* and $\hat{U}$ are the utility value and a normalized utility value, respectively. *P* is a set of points that are produced around hotspot candidates or defined on the frontier.

The literature presents several methods for combining these criteria and providing final utility value. The most common way is to multiply criteria with corresponding weights and then use a summation of these terms to provide the utility value. Equation (4) shows our global utility function:

(4) $$\begin{array}{c} Fitness\left(p\right)=w_{1}\times \left(1-{\hat{U}}_{d}\left(p\right)\right)+w_{2}\times {\hat{U}}_{Sd_{radiation}}\left(p\right)+w_{3}\times {\hat{U}}_{Sd_{obstacle}}\left(p\right)+\\ w_{4}\times {\hat{U}}_{tos_{source}}\left(p\right)+w_{5}\times {\hat{U}}_{pr_{source}}\left(p\right) \end{array}$$

$$\begin{array}{c} \left(w_{1}+w_{2}+w_{3}+w_{4}+w_{5}=1\right) \end{array}$$

where $w_{i\in [1,2,3,4,5]}$ is a corresponding weight.

It is obvious that the process in the exploration will be influenced if the associated weights change. Each weight is related to one criterion, so changing the weights affects the exploration procedure, and also allows the robot to explore different situations.

#### 4.2.3. MCDM Approach Implementation

The MCDM algorithm drives a robot to the point with highest utility value until there are no more points. A specific array maintains all candidate points. The points consist of frontier points and checkpoints that are randomly made around a possible radioactive hotspot for a close-up investigation. The checkpoints are created based on a distance that depends on AR and $d_{s}$ ($d_{s}$ is a user-specified value that is required as a minimum distance between two separated sources. It determines the required accuracy for the radioactive hotspot localization. See Section 6.3.). In addition, if the FOV is not 360 degrees, each checkpoint is split into a few new points with the same location but with different yaw angles to provide multiple views for the robot.

In the next step, the utility function measures the worth of each candidate point based on multiple criteria. The target point is the point with the highest utility. If the chosen point is a point used for approving a radioactive hotspot or for an extension of radiation covering map, the robot stops there and enables the gamma camera. Subsequently, the new possible radioactive hotspots will be added to the candidate list for the next investigation. Finally, through a particular function, we remove some redundant hotspots candidates before the next step. First, we need to remove candidates whose possibility is less than zero. Second, some candidates are falsely identified from sources because of poor AR or inaccuracies in the positioning system, and they must be removed. In Section 4.3, we explain how real radioactive hotspots can be recognized.

### 4.3. The Radioactive Hotspot Validation Method

**Figure 2:**
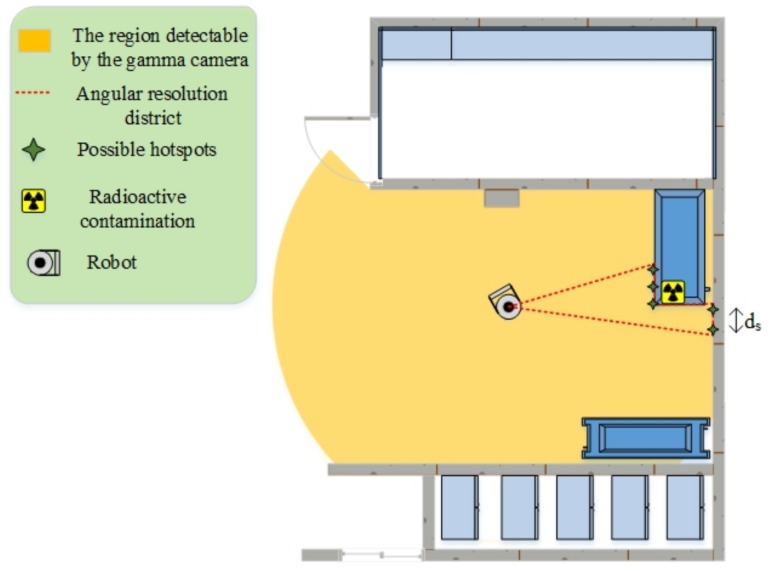
An example showing the possible radioactive hotspots in the contaminated area within a AR district.

As discussed, most gamma cameras have poor ARs (for instance, 20 degrees or more in Table 1). Therefore, if a gamma camera localizes a hotspot far from the robot’s place, there may be other possible sources around it. Most existing algorithms fail to detect sources placed in small regions, especially with no prior knowledge about intensity and source number [53]. More specifically, the red-dotted line in Figure 2 illustrates an imaginary AR district. In this district, the gamma camera will not be able to distinguish separate hotspots, so there might be more than one radioactive source here. This limitation of detectors significantly degrades the accuracy of autonomous exploration; it identifies falsely radioactive hotspots or fails to find real ones. To overcome this limitation and compensate its effects, we designed a novel method to recognize and validate real radioactive hotspots gathered in a narrow region.

Regarding the AR challenge, we set some source candidates in the contaminated area and within the AR district so that the distance between them should be higher than $d_{s}$. Once the gamma camera detects a contaminated region, the source candidates are marked on the region by the laser scanner. The laser scanner helps the robot estimate hotspots’ locations in the robot coordination system. Afterwards, the robot applies particular methods to find real radioactive hotspots among the source candidates; for instance, it can move near them to get a better estimate of the sources through close-up investigations.

In addition, we need to define mechanisms to approve or discard a source candidate. First, each source candidate takes a particular variable that indicates the probability of it being a real source; we already called this possibility. Then, three rules are defined to update the possibilities, as follows:

1. The possibility of each source candidate rises if it is visible by the activated gamma camera. As the robot cannot separately distinguish sources that are placed in the same AR district (red-dotted zone in Figure 2), the possibilities of all candidates on the contaminated AR district increase.
2. The possibility of each candidate source decreases if the candidate source is visible through the gamma camera. The gamma camera will be activated in different locations, so the possibility of fake source candidates will quickly reach zero.
3. The possibility of each source always decreases over time. The rate of this decrease is often very low.

Please note that the rate of increase must be always greater than the rate of decrease. Regarding these rules and rates, the possibility of a real source always increases over time; so source candidates with higher possibility are likely to be a real radioactive source. After finding new source candidates and updating the possibilities, we need an algorithm to approve a real hotspot. This algorithm chooses visible candidates whose possibilities are greater than a defined threshold (We performed a few experiments to find intuitively a suitable threshold, by which the radioactive hotspots localization is consistent, with minimal errors as much as possible. We determined 10 for this threshold.). Next, the algorithm investigates other possible candidates that may exist on the same AR district. If the selected candidate is alone in the AR district, it will be approved. Whenever a candidate’s possibility reaches less than zero, it will be automatically discarded.

## 5. The Architecture of the Autonomous Exploration System

**Figure 3:**
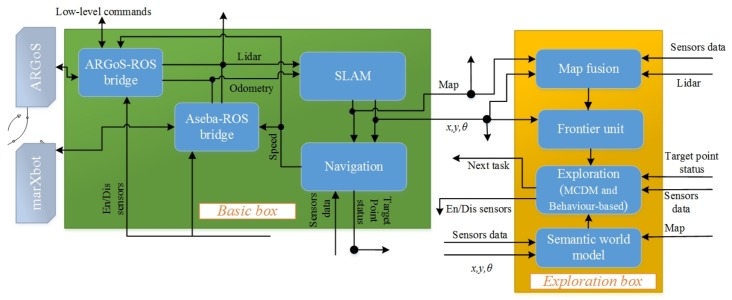
The architecture of the autonomous exploration system for the rescue application.

In this section, we design a search architecture that can implement two different exploration approaches that were described above (Section 4.1 and Section 4.2). Figure 3 represents the architecture of the exploration system that efficiently directs the robot’s exploration and detects and denotes required elements (i.e., radioactive hotspots) in a world model. Below, we discuss the units of the exploration system and their roles in more detail.

*Semantic world model*

It is necessary to have a world model to store and track all elements (radioactive hotspots). After finding an element, we need to add it or update its attributes, such as position and state, to a world model. This model is a global world representation enriched with semantic information over time. It tracks information about elements and their attributes. For instance, the state of an element is modified when the robot approves or discards the element based on additional investigations, and the precision of an element’s location will be improved when a robot moves closer to the element.

The hector_worldmodel stack (http://wiki.ros.org/hector_worldmodel) is designed in the robot operation system (ROS) to collect and integrate semantic attributes of elements in the world model. This package subscribes to messages about radioactive hotspots and then fuses all gathered information together into a single world model.

*Maps and fusion*

In a rescue situation, exploration is often required to collect different data and to build related maps. As mentioned in Section 4.2.1 (*Detectable area*), the robot provides the obstacle and radiation covering maps. The unit map fusion is designed based on the grid_map package of the ROS libraries [54]. This package is a multi-layer map that stores radiation maps, elevation maps, and more. A simultaneous localization and map (SLAM) method provides an occupancy grid map (obstacle map) that is directly stored as the main layer. The second layer is the radiation covering map, which has the same resolution as the main map. It also shows how much of the environment is being covered in the search for radioactive hotspots.

*SLAM*

This unit is embedded in the SLAM unit and provides laser-based 2D SLAM without using odometry; therefore, the system works purely based on the scan-matching method. Scan matching is a process of comparing consecutive scans to each other or to an existing map to determine a robot’s position (http://wiki.ros.org/hector_slam) [55]. The SLAM unit produces the obstacle maps as an occupancy grid map, and it makes the main layer of the grid_map package. In addition, the positions of radioactive sources are stored in the semantic world model based on the global position of the robot; the robot’s position is given by the SLAM unit. Therefore, SLAM is directly linked to the obstacle map and hotspots localization.

*Frontier unit*

**Figure 4:**
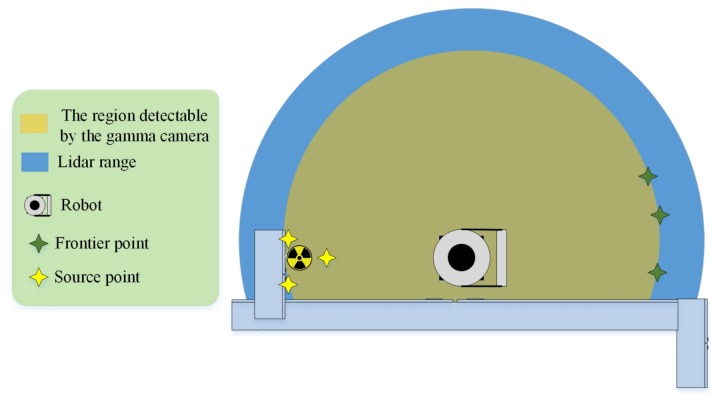
Candidate points are as follows: frontier points on the boundary of the minimum map and points around possible sources. The minimum map is a combination of the obstacle and radiation covering maps.

The frontier algorithm produces points on the boundary between free cells and unexplored areas. It aims to extend the map into new territories by choosing one of these points [21]. The explorations presented in the literature exploited a single map (e.g., occupancy grid map) to create frontier points, whereas our exploration methods need to consider one additional map, as well. Now, the question is how maps have to be considered for the production of frontier points? To answer this question, we fuse the maps together to build a minimum map. The minimum map is the common area between known open spaces of the obstacle and radiation covering maps. Each cell of the minimum map has to be recognized in both maps and must be placed on the free cell of the obstacle map (Figure 4). In addition, we add occupied cells distinguished by the obstacle map to the minimum map; this avoids producing frontier points in inaccessible regions. The minimum map is built in map fusion as the third layer in alignment with the other maps. The frontier unit receives the minimum map as an input and then produces points based on it.

For the behaviour-based approach presented in Section 4.1, the minimum map is equal to only the obstacle map because the radiation covering map will extend when the defined rule for the gamma camera is satisfied. The rule is that if less than k% of the detector coverage area is already covered, the gamma camera will be activated. Thus, we may have some gaps (or holes) in the radiation covering map where the robot never encountered the required condition to start the process of enabling the gamma camera. Some frontier points will constantly appear on the borders of these holes. These frontier points will cause the robot to get stuck. Thus, we consider the minimum map as the obstacle map for the behaviour-based approach.

*Navigation*

The move base unit applied the move_base (http://wiki.ros.org/move_base) package that was developed in ROS to help a robot navigate from its current location to a target point. This package consists of several parts to direct a robot to a destination with a mobile base. The main part is a path planner to produce required trajectories and velocities for robot navigation. The path planner employs both global and local navigation methods to complete a path to the goal. The global path planner finds the minimum cost plan using Dijkstra’s algorithm. The local path planner determines linear and angular velocities based on the Trajectory Rollout [56] and Dynamic Window approaches [57] on a plane. The global and local planners compute costs of traversing based on cost maps (The cost map maintains and updates information about obstacles on an occupancy grid map in order to specify areas about where the robot should not go. (wiki.ros.org/costmap_2d)) and then determines the optimal velocities for look-ahead steps.

*Exploration unit*

The core of this unit is to apply one of the exploration approaches which were presented in Section 4. This unit mainly chooses next point for exploration.

*Aseba_ROS_bridge and ARGoS_ROS_bridge*

These units are middle systems designed in ROS to connect units and low-level hardware. They are also designed to convert raw data from sensors to defined ROS formats or to enable actuators according to high-level ROS commands. In addition, low-level obstacle avoidance with the ability to detect nearby objects and avoid collisions is embedded inside these packages, helping the robot react quickly to environmental changes. This prevents the robots from being damaged if other units (e.g., move base) do not work properly.

## 6. Implementation

### 6.1. Robotic Platform

**Figure 5:**
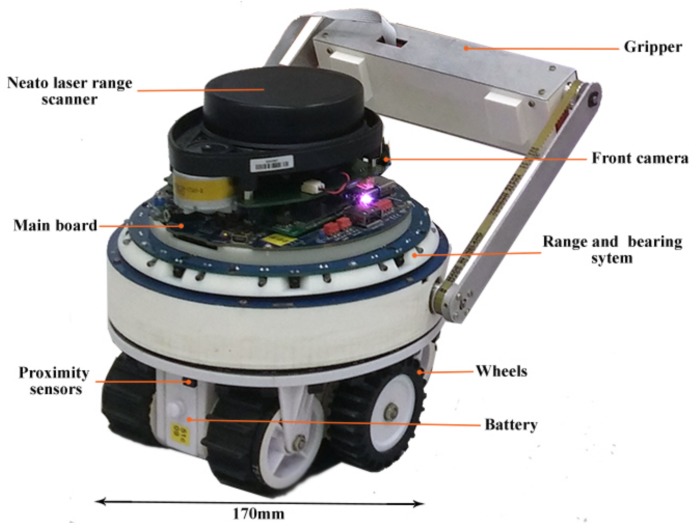
The marXbot, with five of its modules, that was used for our experiments.

As illustrated in Figure 5, a miniature and modular robot called marXbot is used for our experiments [58]. It consists of five independent modules; from bottom to top, they are as follows: a base module for mobility of the robot, a gripper for manipulating the objects (but we do not use this module for our experiments), the range and bearing system for discovering the relative position of other surrounding robots, the main board and front camera, and a distance scanner module to do SLAM.

### 6.2. Simulator

ARGoS is used to model and simulate large-scale swarms of mobile robots. It was initially developed under the EU-funded Swarmanoid project (www.swarmanoid.org.) that aimed to study coordination between different kinds of robots. One of these three robots was the marXbot. With ARGoS, we simulate our exploration experiments using the marXbot [59]. We have chosen ARGoS for performing our experiments, because ARGoS was developed based on the marXbot to run robotic experiments; in addition, it is fast and customizable.

### 6.3. Gamma Camera and Radioactive Sources

In conventional gamma cameras, an incident gamma-ray is scattered through a top layer and then absorbed by a bottom layer. The axis and angle of each incident gamma-ray can be an imaginary cone, so the intersections of the rebuilt cones show the source location. In addition, the incident gamma-ray allows us to calculate the energy of the radioactive hotspots [60]. Each radioactive hotspot may emit gamma-rays in the different energy range (from a few keV to 10 MeV) and the imaging performance of the gamma camera depends on what types of radioisotopes are placed in an environment. According to the literature, a gamma camera shows different performance in terms of the acquisition time and AR at different energies ranging [61]. In addition, a gamma-ray energy spectrum that is produced based on these collected gamma-rays can be used to identify radioactive hotspots [60].

**Figure 6:**
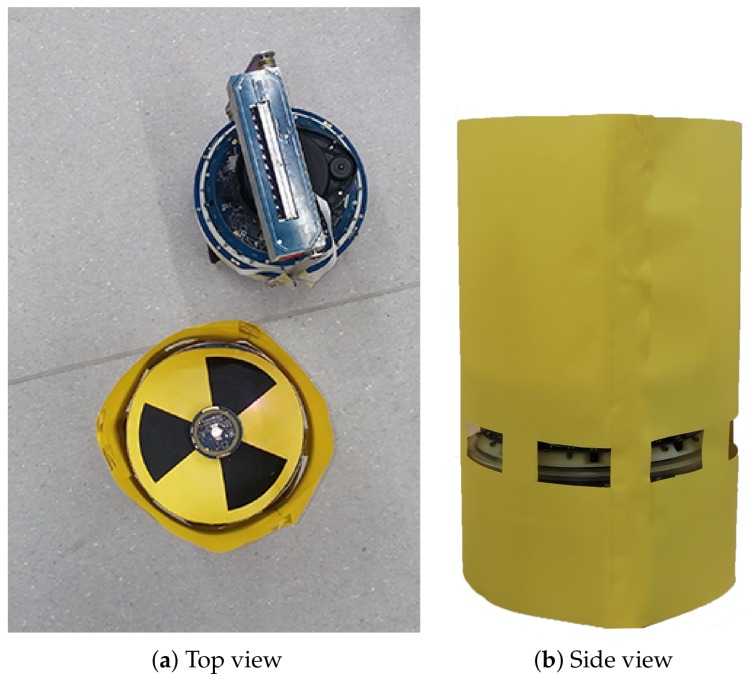
The source is another marXbot in which the range and bearing system acts as a radioactive source.

However, to comply with our requirements for providing a safe test-bed in the laboratory, we could not use real radioactive materials. Therefore, we replaced them with materials that have similar behaviours. In the real-world setup, sources are other marXbots, in which case the range and bearing systems send infrared rays instead of radioactive waves (Figure 6). All sources are supposed to emit radioactive waves with identical intensity and frequency; the intensity is inversely proportional to the square of the distance. An object that is close to a source absorbs 0.01 $\frac{Gy}{s}$ radiation. The related gamma camera is also modelled alongside the new radiation matter. The range and bearing system of the main marXbot acts as a detection system. It can estimate its distances to sources and then compute the corresponding intensity. In the simulation, sources are lights with the same intensity, which are likewise inversely proportional to the square of the distance. A series of light sensors is defined around the robot to model a directional detector.

## 7. Experiments, Evaluations, and Discussions

The two methods proposed for autonomous exploration are implemented and extensively tested in simulation (ARGoS). We also performed a series of real experiments with the marXbot to validate the simulation results.

**Figure 7:**
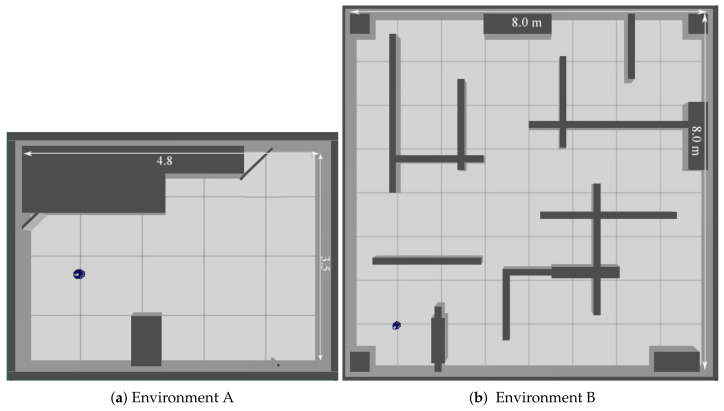
A top view of the environments (A, B) used in the simulation.

Three types of environments are considered in the experiments. The first environment that is introduced in Figure 7a is named environment *A*; if it is filled with debris and obstacles, we named it environment *C* that is similar to Figure 7a. We also considered another different environment with dimensions of 8 m by 8 m with many internal walls; we named it environment *B*.

We also change the gamma camera parameters to discover how they impact exploration performance. The values in Table 2 are assumed for FOV and AR. The best gamma camera has a FOV of 360 degrees and an AR of 2.5 degrees. We first change the FOV from 360 to 45 degrees to see the effects on exploration performance. Accordingly, we change AR from 2.5 to 30 degrees to study the related effects on performance. Each state (each column in Table 2) was repeated ten times for the three types of environment.

The MCDM approach operates based on a utility function that consists of criteria and corresponding weights. We can achieve different exploration behaviours by changing the weights. Typically, a weight is assigned to the criteria to indicate its relative importance in MCDM [62]. This is a design choice, done by the designer to indicate which criteria have which importance. Indeed, MCDM is not a method to find the best exploration solution but a flexible and general method to combine criteria [28]. Therefore, for choosing the weights of MCDM, we manually defined different weights based on what we decided to be more or less important and then ran exploration experiments; later we came back to choose the weights based on the performed experiments. Table 3 reports the weights of Equation (4) (utility function) that have been selected among the proposed weights and assigned manually. The set of weights consists of two steps as follows: first when we have candidate sources on the environment, and second when there is no candidate source or the robot has not detected a candidate source yet. The termination conditions for exploration are: if the exploration time exceeds a user-defined time or the robot entirely scans an environment to complete all maps (i.e., radiation covering and obstacle maps). The radiation covering map has the same resolution as the obstacle map; in both cases, each cell is 0.04 m (Selecting a big cell size will reduce the computational effort of SLAM but the map accuracy and robot localization will become worse. In contrast, a small cell size will have the opposite effects. Thus, the cell size affects the exploration time, accuracy of the maps, and localization precision of detected hotspots.). In the behaviour-based approach, the robot always checks how much area the gamma camera has already covered. If it is less than a defined threshold, for our case 50%, the robot stops to record a new radiation image. Ten trials were performed for each exploration method in simulation, and two radioactive hotspots were randomly placed in the environment for each trial.

### 7.1. Comparison of the Two Exploration Approaches

**Figure 8:**
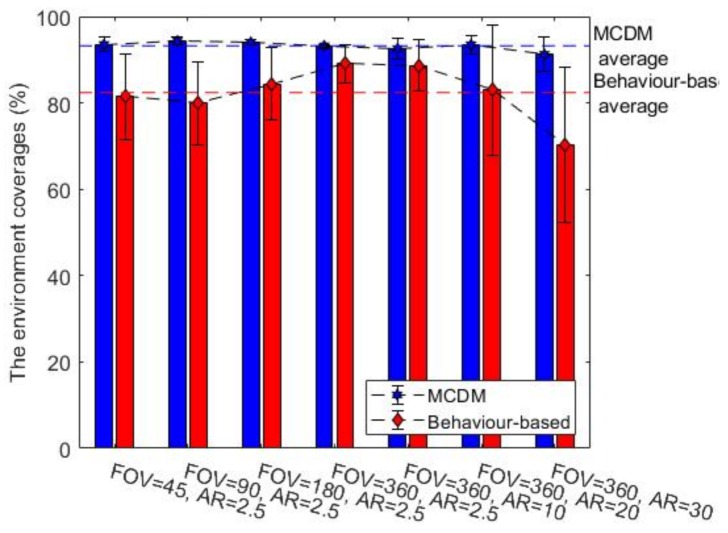
The obstacle map coverage percentages for the environment B, average over ten trials.

**Figure 9:**
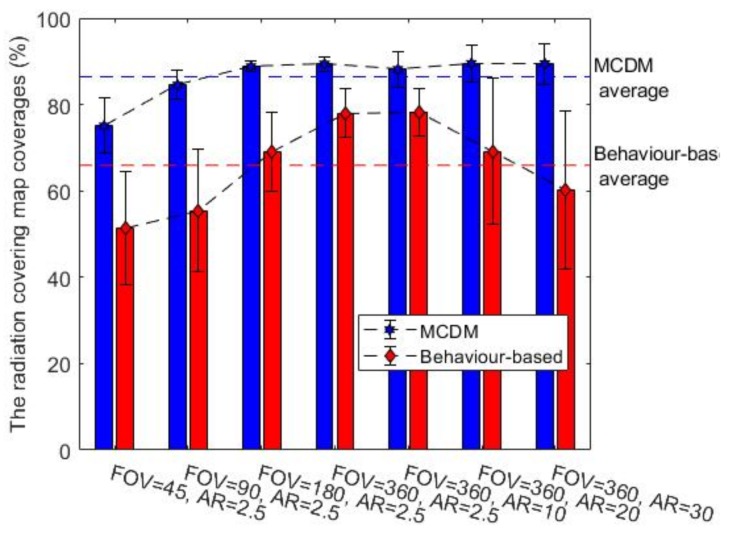
The radiation covering map coverage percentages for the environment B, average over ten trials.

**Figure 10:**
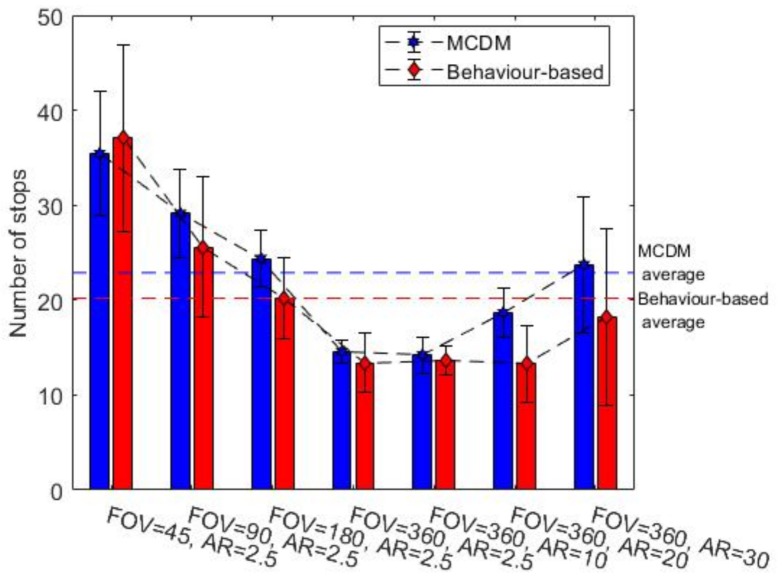
The number of stops required for the gamma camera to give a radiation image in the environment B, average over ten trials.

In this section, we study and compare the behaviour-based and MCDM approaches in an environment with radioactive contamination hazards. The final obstacle and radiation covering map coverage percentages are represented in Figure 8 and Figure 9 for environment B, respectively. (In order to avoid repetition, we show only the results of environment B as they are representative of the other observed results. This environment is more complex and better highlights the interesting aspects.) The middle bar shows the best gamma camera (FOV of 360 and AR of 2.5 degrees), and the other bars show degraded gamma cameras. The obstacle map coverage percentage is almost constant for MCDM, and coverage is about 10% higher than the behaviour-based approach. For building the radiation covering map, MCDM worked significantly better than the behaviour-based approach. Additionally, the radiation chart shows the behaviour-based approach discovered about 20% fewer regions than the MCDM method. In terms of the termination conditions, the graphs of the obstacle map coverage percentages show that the robot could not entirely explore the environment in the behaviour-based approach within the given time. Figure 10 shows the number of times the robot stops for recording radiation images. As we discussed in Section 4.2, the radiation image could be applied to extend the maps or check the possible radioactive hotspots. The stop chart shows that the robot stopped more when it had a gamma camera with low FOV or poor AR. The environment included two radioactive sources that were randomly placed. Table 4 shows that the likelihood of hotspot detection in the behaviour-based approach was 93% and 50% for the first and second radioactive hotspots, respectively, but the likelihoods are near 100% in MCDM for both hotspots. In addition, the number of stops in MCDM is almost less than behaviour-based; it shows that the robot could find more radioactive hotspots by fewer stops.

The comparison between the two methods also shows that the behaviour-based approach is more heavily influenced by the gamma camera’s parameters. The obstacle coverage percentages in the MCDM approach are almost constant over different values of the gamma camera’s parameters, but the percentages decrease in the behaviour-based approach when the parameters move away from the ideal values. Changing the FOV could impact the behaviour-based approach more than the MCDM approach in terms of the radiation coverage percentages. The AR variation also affected the radiation coverage percentages in the behaviour-based approach while it had little impact on the coverage percentages in the MCDM approach.

In addition, the comparison between two exploration methods allowed tentative evaluation of the algorithm’s value for rescue. According to the results, the MCDM approach is a more efficient algorithm than the behaviour-based approach for the same time. The obstacle and radiation covering map coverage percentages grew 30% faster in the MCDM approach than in the behaviour-based approach. The MCDM approach could localize almost all radioactive hotspots, but the behaviour-based approach missed some radioactive sources. Moreover, it seems that the MCDM approach is more robust with different types of gamma cameras, while the behaviour-based approach is more heavily influenced by the gamma camera’s parameters.

### 7.2. Further Studies on the MCDM Approach

In the previous section, we showed that the MCDM approach seems a more efficient exploration algorithm than the behaviour-based approach. In this section, we study the MCDM approach in more detail. We also continue to study the gamma camera’s parameters and show how they impact exploration and detection. The values in Table 2 are given for FOV and AR.

In addition to studying the effects of the gamma camera’s parameters on exploration, we investigate which kind of gamma camera is more appropriate for radioactive hotspot detection and localization: the gamma camera with a FOV of 360 degrees and an AR of 30 degrees (high FOV and poor AR) or the gamma camera with a FOV of 45 degrees and an AR 2.5 degrees (low FOV and good AR). The commercial gamma cameras reported in Table 1, either with the high FOV and poor AR (e.g., polaris-H) or low FOV and good AR (e.g., iPIX) are usually available. Therefore, answering this will indicate which gamma camera is the best choice for exploration using mobile robots.

**Figure 11:**
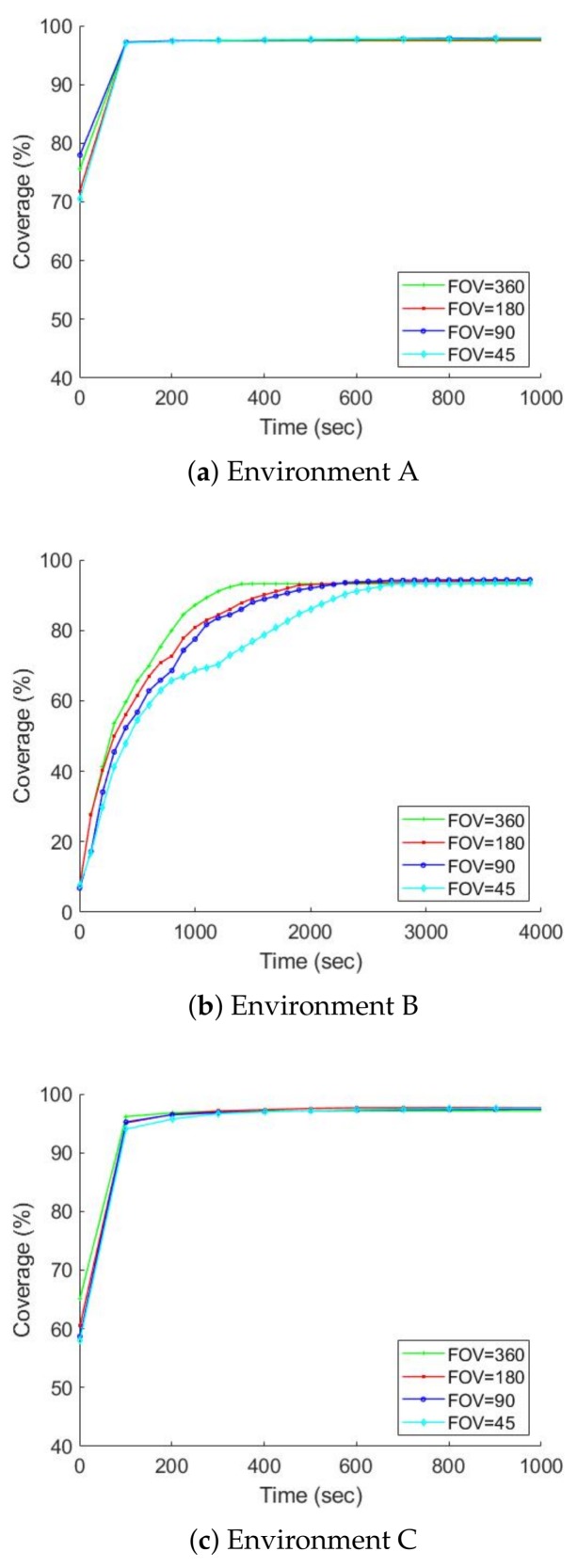
The obstacle map coverage over time based on a set of FOV, AR is equal to 2.5 degrees, average over ten trials.

**Figure 12:**
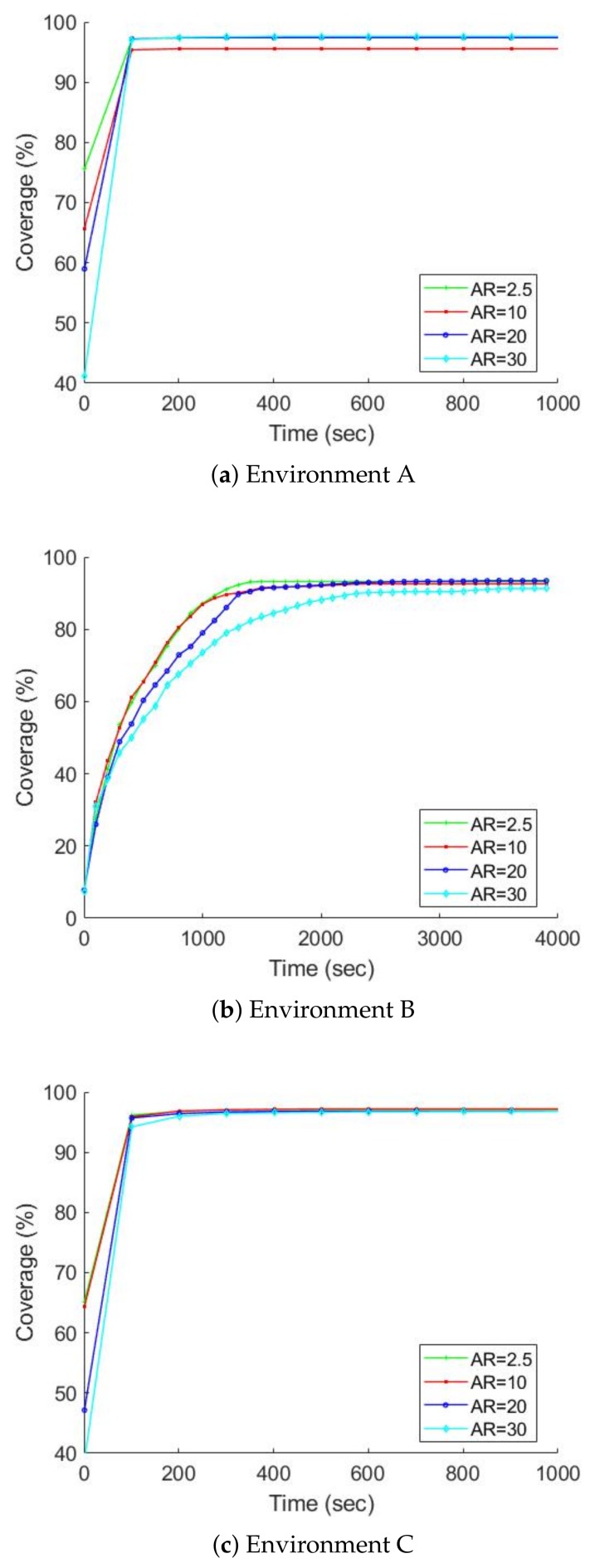
The obstacle map coverage over time based on a set of AR, FOV is equal to 360 degrees, average over ten trials.

**Figure 13:**
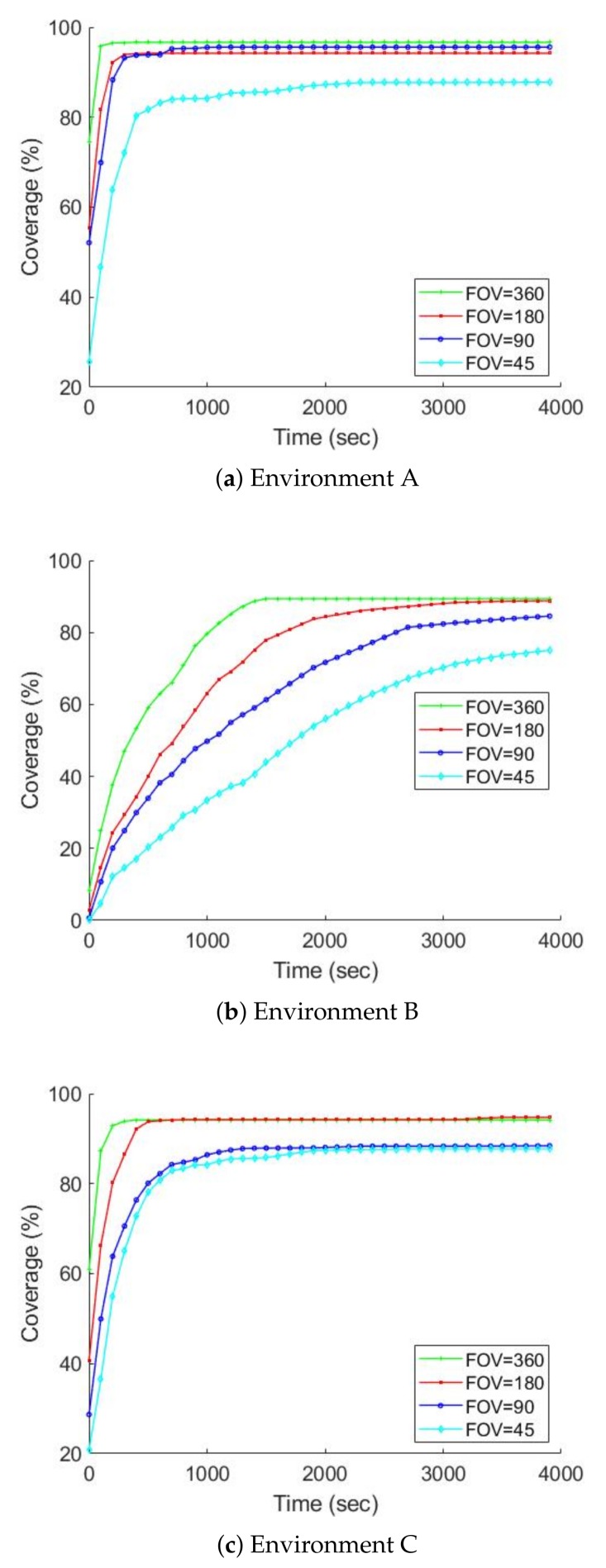
The radiation covering map coverage over time based on a set of FOV, AR is equal to 2.5 degrees, average over ten trials.

**Figure 14:**
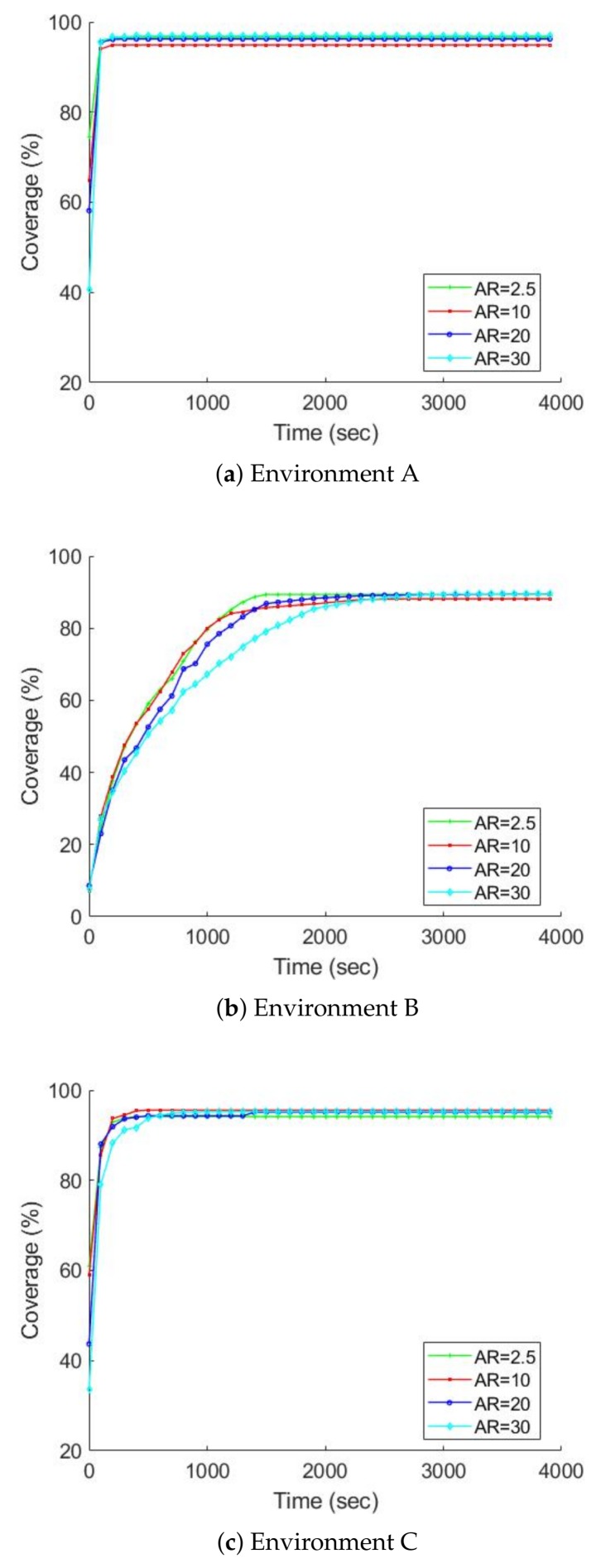
The radiation covering map coverage over time based on a set of AR, FOV is equal to 360 degrees, average over ten trials.

Figure 11 and Figure 12 chart the progress of the obstacle coverages based on a set of FOV and AR over time; Figure 13 and Figure 14 show the radiation coverages based on a set of FOV and AR over time. They show how the complexity of the environment influences the growth of the map. The surface area of environment B is four times bigger than A (or C), but the graphs of B took ten times as long to reach a steady state. It seems that the internal walls effectively decreased the rate of map extension because they blocked the laser scanner’s range.

The second important factor for the growth of the map is the parameters of the gamma camera. Because of the use of the maximum FOV scan (360 degrees), the fastest growth occurs in all three environments, especially with the radiation covering map. The graphs of environment B present the effects of these parameters; Figure 13 shows that if we degrade the gamma camera from a FOV of 360 degrees to 45 degrees, the coverage speed will decrease approximately 50%. In contrast, if we degrade the gamma camera from an AR of 2.5 degrees to 30 degrees, the coverage speed will decrease about 10%, as illustrated in Figure 14.

**Figure 15:**
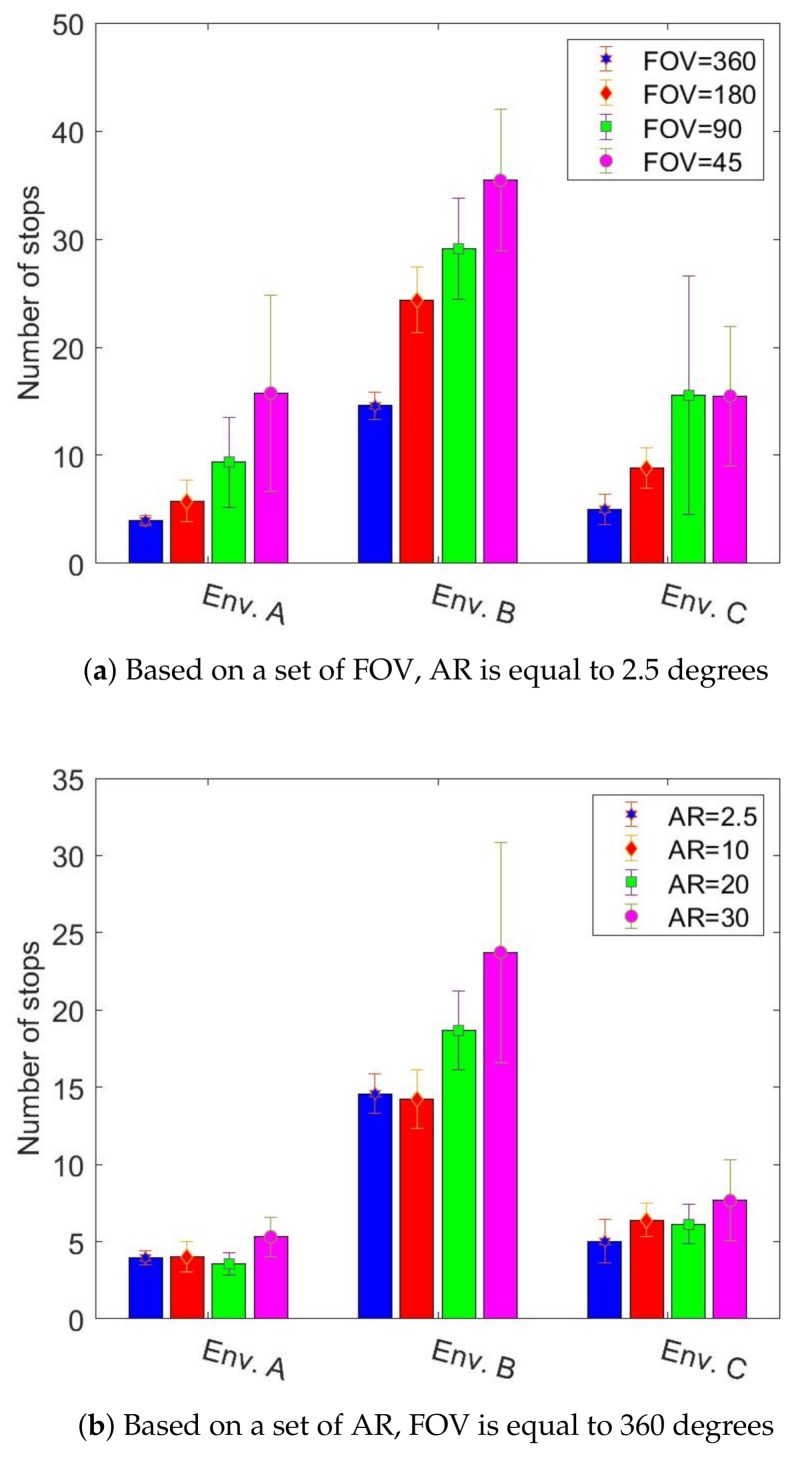
The number of stops required for the gamma camera to give a radiation image in MCDM, average over ten trials.

**Figure 16:**
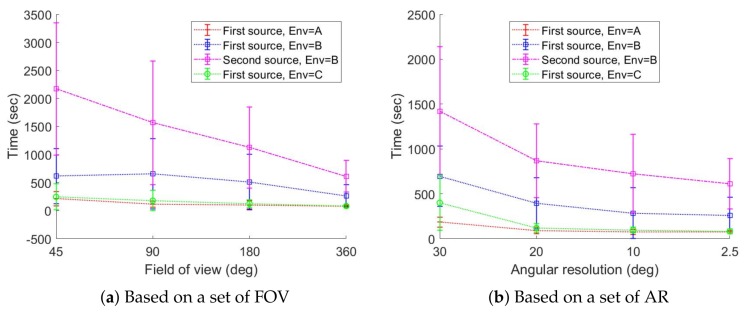
The time required for radioactive hotspot localization, average over ten trials.

Figure 15 shows the number of times the robot stops for recording radiation images. The graphs show that the robot stopped more when it had a gamma camera with low FOV or poor AR. The robot attempted to stop more for low FOV than for poor AR. The graphs in Figure 16 show the time needed to localize radioactive hotspots for a set of FOV and AR. The graphs show that the localization time decreased with improvements to the gamma camera. Likewise, in these graphs, we can see that the gamma camera with a FOV of 45 degrees performed worse than the gamma camera with an AR of 30 degrees.

**Figure 17:**
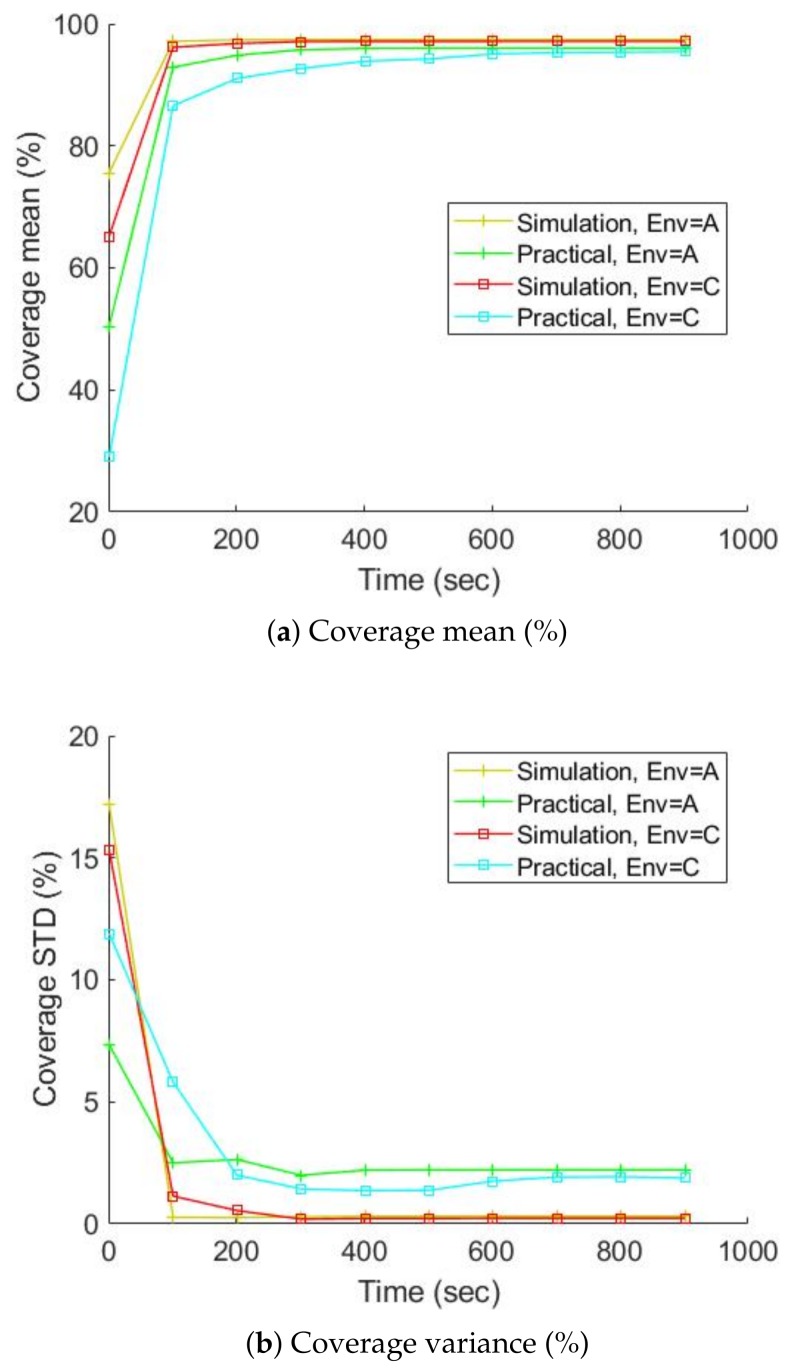
Comparison of the coverage of the obstacle map from the simulation, and from using the real robot, average over ten trials.

**Figure 18:**
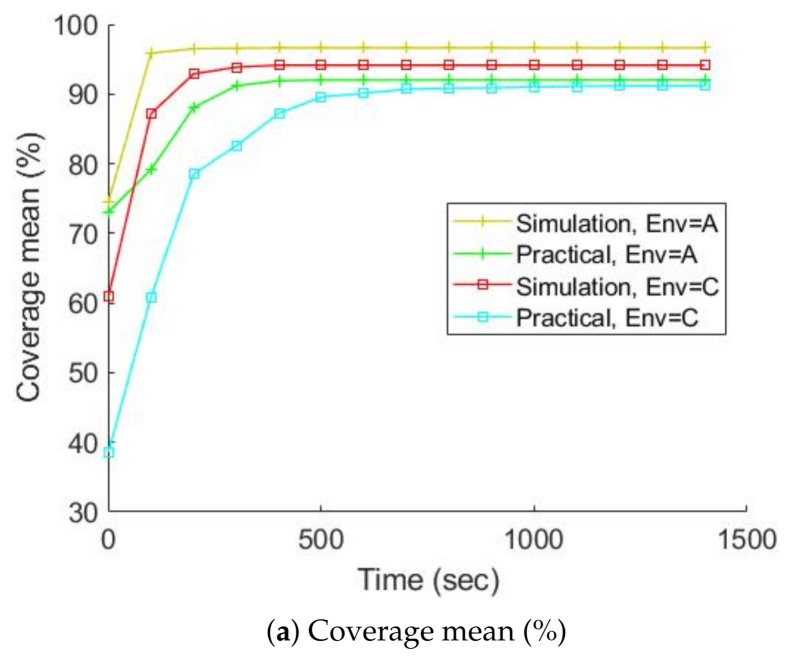

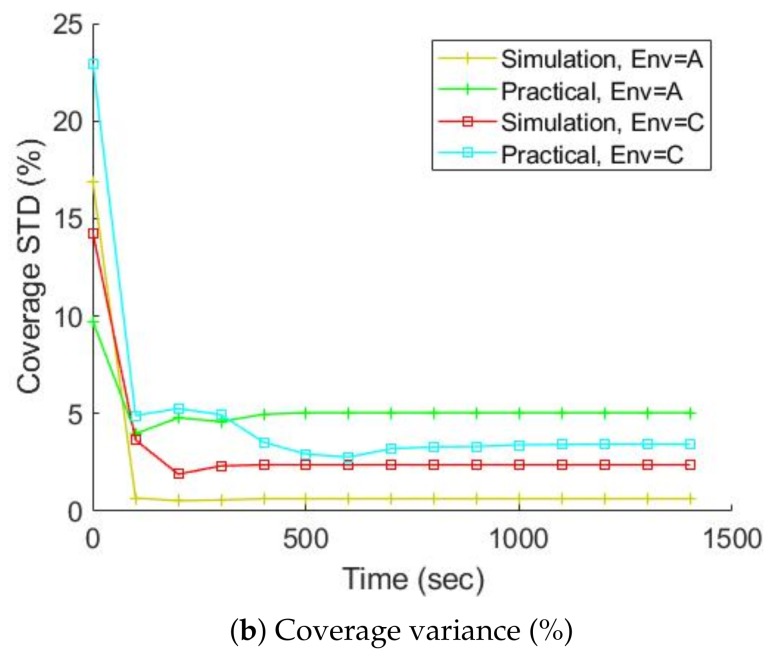
Comparison of the coverage of the radiation covering map from the simulation, and from using the real robot, average over ten trials.

**Figure 19:**
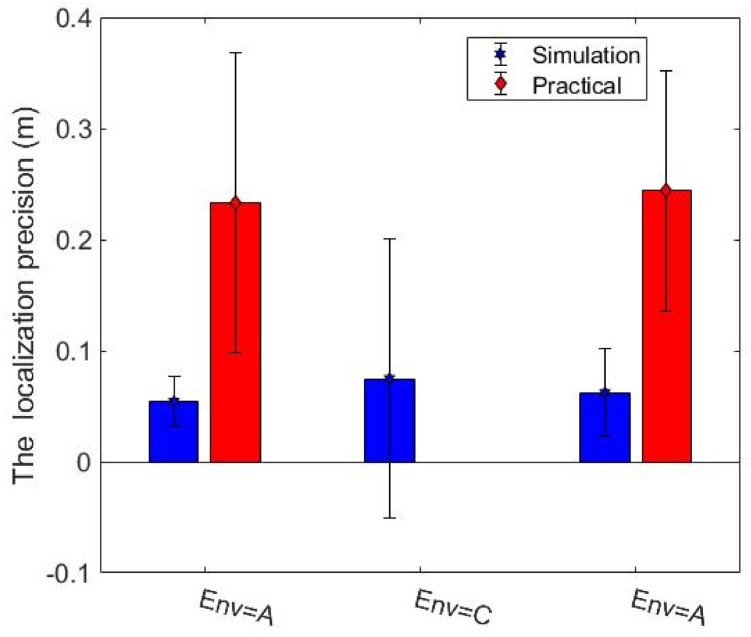
The localization precision of detected hotspots.

The studies done in simulation were validated by experiments performed on the real robot (marXbot). The specification of the gamma camera was reported in Table 1, but we could only run experiments for a real robot with a FOV of 360 degrees and an AR of 2.5 degrees. Environments A and C were considered, and ten trials were run for each of them. Figure 17 and Figure 18 address the difference between the obstacle and radiation covering map coverage in the simulation and with the real robot, respectively. The practical coverages from the real experiments are slightly lower than the simulation coverages. There might be some small unknown regions near the walls that could have trapped the robot. In creating our exploration algorithms, we considered using a border around the walls to prevent the robot from getting stuck. This border was thicker in the practical experiments because of the inaccuracies of the positioning and mapping systems. Therefore, the real robot ignored more unknown small regions, so the practical coverage is slightly lower than the simulation coverage. The localization precision of detected hotspots is also investigated. The localization precision is severely affected by the SLAM precision because the robot has to determine the locations of hotspots on the map built by SLAM. SLAM produces maps and gives the robot’s position, so SLAM errors affect the accuracy of the built maps and localization precision of detected hotspots. Figure 19 shows the localization precision of detected hotspots in real and simulated experiments. The precision in the real environment is worse than simulated experiments due to more uncertainty in the real experiments.

So, which kind of gamma camera is more appropriate for radioactive hotspot detection and localization: a gamma camera with high FOV and poor AR or a gamma camera with low FOV and good AR? A gamma camera with higher FOV and poor AR is more suitable for exploration and radioactive hotspot localization because it can cover more areas for the same time. Please note that we made these conclusions based on one or two radioactive sources. Certainly, the behaviours of some graphs would change if the number of sources increased. As a result, for the time being, the conclusion about the best choice of gamma camera is valid only for environments with a low density of radioactive sources. Further experiments will be needed to investigate the best choice in environments with many radioactive sources.

## 8. Conclusions

In this research, we studied the behaviour-based and MCDM exploration approaches for the autonomous mapping and localization of elements in an environment after a nuclear disaster. These methods were developed for mobile robots that use gamma cameras for discovering radioactive hotspots. As the limitations of gamma camera technologies (long acquisition time and poor AR) were two main challenges for available autonomous exploration methods, we adapted and developed effective exploration algorithms to overcome these limitations. Both methods have addressed an efficient exploration; the robot explores the environment and localizes hotspots without scanning the entire environment.

We have shown that how changing the gamma camera parameters (FOV and AR) can impact exploration performance. In addition, we have shown the number of stops required for the gamma camera to give a radiation image after the acquisition time, and the number of stops affects the total completion time. From our results, we also conclude that MCDM is a more effective method for exploration and mapping of radioactive sources than the behaviour-based approach. In particular, MCDM reported significant improvements in more complex situations (e.g., environment B) where MCDM can demonstrate its superiority by making good decisions. We have also shown that the gamma camera with higher FOV and poor AR is more suitable than a gamma camera with lower FOV and good AR in environments with low density of radioactive sources. Moreover, the poor AR limitation of detectors often resulted in the identification of false radioactive hotspots or the inability to find real ones. To overcome this limitation and compensate its effects, we designed a new method to discover and validate the locations of radioactive hotspots among many candidates in the environment.

As part of future work, we aim to develop the presented methods based on multi-robot systems. Multi-robot systems allow robots to share their information and then accomplish exploration tasks faster than a single robot. Other advantages of multi-robot systems like parallelism, robustness, scalability, and fault tolerance can also improve radioactive hotspot localizations. Moreover, thanks to promising results, these methods can be generalized and applied to mobile robots equipped with this kind of tardy detectors (i.e., a gamma camera), for instance, to detect and localize objects in dark environments by cameras.

## Figures and Tables

**Table 1:** Specifications of the selected commercial directional detectors.

Detectors	Type	Field of view (FOV) (deg)	AR (deg)	Acquisition Time (s)	Weight (kg)
Polaris-H [45]	Compton (CZT)	360	20–30	30<	4.04
HSL-Lite [46]	Coded mask and dynamic imaging mask	60	<10	900<	6.5
Toshiba [47]	-	60	-	-	9.8
iPIX [48]	GAMPIX coded mask	45–50	2.5–6	1<	2
AISense [44]	Hotspot locator (without camera)	360	-	0.1	2.2

**Table 2:** The values are assumed for the parameters of the customized gamma camera.

States	1	2	3	4	5	6	7
FOV (deg)	360	360	360	360	180	90	45
AR (deg)	30	20	10	2.5	2.5	2.5	2.5

**Table 3:** The weights were applied in the utility function.

Condition	$w_{1}$	$w_{2}$	$w_{3}$	$w_{4}$	$w_{5}$
With a candidate hotspot	0.3	0.1	0.05	0.3	0.25
Without any candidate hotspot	0.3	0.6	0.1	0	0

**Table 4:** The likelihood of hotspot detection, average over ten trials.

Method	First Source (Ave.)	Second Source (Ave.)
Behaviour-based	93%	50%
MCDM	100%	97%

## Abbreviations

The following abbreviations are used in this manuscript:

**AR**	Angular Resolution
**FOV**	Field Of View
**MCDM**	Multi-Criteria Decision Making
**ROS**	Robot Operating System
**SLAM**	Simultaneous Localization And Mapping
**UAV**	Unmanned Aerial Vehicle
